# Toxicological Evaluation of Piceatannol, Pterostilbene, and ε-Viniferin for Their Potential Use in the Food Industry: A Review

**DOI:** 10.3390/foods10030592

**Published:** 2021-03-11

**Authors:** Concepción Medrano-Padial, Ana Isabel Prieto, María Puerto, Silvia Pichardo

**Affiliations:** Area of Toxicology, School of Pharmacy, Universidad de Sevilla, C/Profesor García González n°2, 41012 Seville, Spain; cmpadial@us.es (C.M.-P.); mariapuerto@us.es (M.P.); spichardo@us.es (S.P.)

**Keywords:** food control, piceatannol, pterostilbene, ε-viniferin, toxicological studies

## Abstract

The application of stilbenes in the food industry is being considered because of their biological activities. Piceatannol, pterostilbene and ε-viniferin have awakened the industry’s interest. However, before they can be commercialized, we must first guarantee their safety for consumers. The present work reviews the toxicological studies performed with these stilbenes. A wide variety of studies has demonstrated their cytotoxic effects in both cancer and non-cancerous cell lines. In contrast, although DNA damage was detected by some authors, in vitro genotoxic studies on the effects of piceatannol, pterostilbene, and ε-viniferin remain scarce. None of the three reviewed substances have been evaluated using the in vitro tests required by the European Food Safety Authority (EFSA) as the first step in genotoxicity testing. We did not find any study on the toxic effects of these stilbenes in vivo. Thus, more studies are needed to confirm their safe use before they can be authorized as additive in the food industry.

## 1. Introduction

During the last decades, the interest in polyphenolic phytochemicals has increased markedly due to their beneficial properties [1]. Natural polyphenols are abundant in fruits, vegetables, whole grains, and foods and beverages derived from them such as chocolate, wine, olive oil, or tea; thus making it the most important phytochemical present in the human diet [2]. These compounds are highly diversified and comprise several subgroups of phenolic compounds ranging from simple substances, including phenolic acids and stilbenes, to complex polymerized molecules, such as tannins [3].

**Figure 1:**
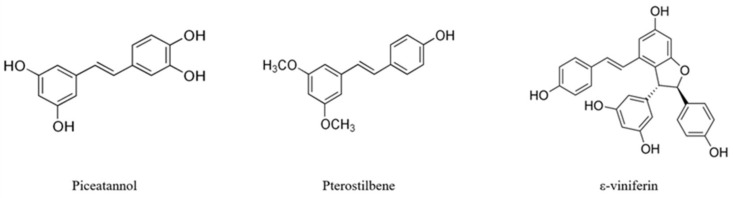
Chemical structures of piceatannol, pterostilbene, and ε-viniferin.

Natural stilbenes are secondary metabolites produced by plants to protect themselves against stressful conditions such as ultraviolet irradiation, excessive heat and fungal or bacterial infections [2]. Structurally, stilbenes are characterized by the presence of a 1, 2-diphenylethylene nucleus [4] and they can be found in E, or trans, and Z, or cis configurations, the trans form being the one that exhibits more potent pharmacological activities [5,6]. Moreover, these compounds exist as monomers, such as resveratrol, piceatannol, or pterostilbene, and oligomers, like ε-viniferin [1] (Figure 1). 

There are more than 400 natural stilbenes reported, however, they are only distributed in a small and heterogeneous group of plants such as wine grape (*Vitis vinifera*), peanut (*Arachis hypogaea*), and some tree species (*Pinus* and *Picea* genera) because stilbene synthase, the key enzyme involved in stilbene biosynthesis, is not ubiquitously expressed [4]. In general, the highest amount of stilbenes is found in grapes and wine derivatives. However, data related with the available concentrations of these compounds from different sources is very scarce because it depends on the variety of grapes, agricultural and environmental factors (soil, temperature, pathogen attack) and the complexity of the qualitative and quantitative analysis of different stilbenes [7,8]. Moreover, residues produced during wine making such as grape pomaces and other grape juice solids contain high polyphenol concentrations and are important sources of many stilbene compounds, which is interesting because sustainability in food production has become an area of utmost importance [9].

These compounds have been widely used in the manufacture of industrial dyes, laser dyes, optical brighteners, phosphors, and scintillators [5]. However, in recent years, stilbenes and their analogues have awakened the interest of the scientific community due to their diverse spectrum of biological applications such as anticarcinogenic, antiproliferative, antiangiogenic, antimicrobial, antileukemic, anti-inflammatory, antioxidant, antimutagenic, and antigenotoxic agents, and as a vasodilator [2,10,11], among others [6]. Furthermore, numerous studies have indicated a positive effect of these compounds against diseases related to oxidative stress including cancer, cardiovascular, and autoimmune diseases [12], aging [13] and neurodegenerative pathologies [1]. These preventive effects of stilbenes are mainly due to their antioxidant activity by scavenging free radicals, but recent lines of evidence suggest that they can also interact directly with multiple intracellular signaling cascades involved in the development of numerous pathologies [2]. Moreover, the use of stilbenes as natural preservatives has recently become an area of growing interest because synthetic additives are increasingly rejected by consumers, who now give preference to ingredients from natural sources [14]. 

These new applications of stilbenes in the food industry have caused some concern regarding their safety for consumers since the intake of these stilbenes may increase. In this sense, the estimate human consumption of stilbenes depends on many factors such as the type of diet and food processing, leading to a large variability of the exposure scenario [7,8]. Then, a toxicological evaluation is required by the European Food Safety Authority (EFSA) prior to their commercial use. The first approach to determining the toxicity effects of any compound should be the use of in vitro cytotoxicity tests to define basal cytotoxicity, which is directly related to cell death induction. Following the EFSA’s Panel on Food Additives and Nutrient Sources added to Food (2012) guidelines, a step-wise approach is recommended for the evaluation of data on the genotoxic potential of these compounds, starting with a basic battery of two in vitro tests, comprised of the bacterial reverse-mutation assay (Ames test, OECD 471) and the micronucleus test (OECD 487). In the case of inconclusive, contradictory, or equivocal results, it may be appropriate to conduct further in vitro testing [15]. Additional in vivo studies are also needed before its commercialization. These studies include genotoxicity, toxicity (subchronic, chronic, and carcinogenicity), reproductive, and developmental toxicity testing, etc. [15,16]. Therefore, besides their well-known beneficial effects, stilbenes may also exhibit toxic effects. The toxicity of trans-resveratrol, the most extensively studied stilbene, has been evaluated by other authors [17,18]. This stilbene has been categorized as GRAS (Generally Recognized as Safe) by the US Food and Drug Administration (FDA) [19]. In addition, trans-resveratrol with ≥99% (*w/w*) purity has obtained EFSA approval as a novel food [16]. In this sense, because of its safe status, properties, and consumer acceptance, some resveratrol derivatives such as piceatannol, pterostilbene, and ε-viniferin have recently piqued the interest of industries [20]. However, very few reports have analyzed the toxicity of these derivatives. In this regard, the aim of the present work was to review and provide a compilation of the scientific publications focused on in vitro and in vivo toxicological studies of piceatannol, pterostilbene, and ε-viniferin carried out to date.

## 2. Cytotoxicity in In Vitro Studies Performed with Stilbenes

Cytotoxicity studies are the first approach in defining the toxic effects of any compound since they are simple, fast, and have a high sensitivity. These assays define the basal toxicity related to cell induction and are a first step in evaluating the safety of the tested molecules [21]. In this regard, the results of the cytotoxic and morphological studies carried out thus far in piceatannol, pterostilbene, and ε-viniferin are shown in Table 1, Table 2, Table 3, Table 4, Table 5 and Table 6. It is interesting to point out that, although stilbenes have been used in traditional medicine since ancient times [22], most of the studies concerning the cytotoxicity of these stilbenes have been published recently, between the years 2001 and 2020. 

The most frequently used biomarker to assess the cytotoxic effects of these stilbenes is the 3-(4, 5-dimethylthiazol-2-yl)-2, 5-diphenyltetrazolium bromide (MTT) assay. It measures the mitochondrial metabolic rate and indirectly reflects the viable cell number [23]. This is one of the most popular techniques for screening the effects of compounds on cultured cells. However, some stilbenes exhibit MTT-reducing activity which can lead to inaccurate readings [23]. In this sense, several authors have used alternative biomarkers of cell viability such as the trypan blue dye exclusion test (TBET), cell counting kits (CCK), water soluble tetrazolium salt-1 (WST-1), Sulforhodamine B (SRB) assay, neutral red uptake (NRU), lactate dehydrogenase (LDH) activity, 2-(2-methoxy-4-nitrophenyl)-3-(4-nitrophenyl)-5-(2,4-disulfophenyl)-2H-tetrazolium (WST-8) assay, 2,3-bis-(2-methoxy-4-nitro-5-sulfophenyl)-2H-tetrazolium-5-carboxanilide (XTT) assay or automated cell counting (CC108).

Additionally, it seems that some polyphenols induce cytotoxicity in a cell type-selective manner [24]. In relation to the experimental models selected, it should be emphasized that most experiments have been performed in human cancer cell lines. This is because their main purpose was to assess the potential of these compounds as anticancer agents, since these stilbenes can modulate cellular oxidative stress levels and induce DNA damage. Moreover, these compounds, in combination with chemotherapeutics, can have chemoprotective and synergistic effects [25,26], which is of great interest for potential therapeutic uses. However, although stilbenes are not known to exhibit toxicity toward normal cell lines [27], cytotoxic effects have also been recorded after piceatannol, pterostilbene, and ε-viniferin exposure in non-cancer cell lines. The studies performed on these cells are far fewer and the results differ depending on the stilbene tested, cell lines used, assay performed, and exposure conditions. In this sense, it is also important to evaluate the effects of these stilbenes in normal cell lines to assert their safety before they can be used for industrial use. In general, although high concentrations were required to obtain an IC_50_ value up to 400 µM, a decrease in cell proliferation was recorded in a time- and dose-dependent manner. This effect was even observed at lower concentrations such as 30 µM for piceatannol [28], 40 µM for pterostilbene [29], and 20 µM for ε-viniferin [30]. These findings are relevant since non-cancerous cells are usually more sensitive, and the results could be easily extrapolated to human systems [31]. 

The cytotoxic effects of piceatannol are described in (Table 1). Lymphoma cells such as HL-60 cells [24,32,33,34,35,36], L1210 cells [35,37], or K562 cells [35,36,38] have been the most extensively used to study this stilbene, followed by melanoma cells [39,40,41,42], and colon [37,43,44], prostate [45], or liver [24] cancer cell lines. Contradictory cytotoxic results have been obtained since different methods and cell lines have been used. In general, most of the authors stated that piceatannol has cytotoxic effects in a dose- and time-dependent manner in cancer cells lines at concentrations between 20–100 µM after 24 and 48 h of exposure. Moreover, cytotoxic effects have also been reported in non-cancerous cells treated with piceatannol [28,32,46]. Similar to that observed in cancer cells, concentrations from 30 µM affected the cell viability of normal HUVEC cells after 48 h [28]. In contrast, higher concentrations were necessary to observe toxic effects in the two non-tumor oral human cells, HGF (gingival fibroblast) and HPC (pulp cells), reaching CC_50_ values at 364 µM and 414 µM after 24 h of exposure [32]. The results show high variability as a function of the non-cancerous cell line model selected for the test. The toxicity of this stilbene seems to be related to the ortho-dihydroxyl groups on the phenyl ring, also known as catechol. This is in agreement with other authors who stated that the hydroxylation of resveratrol in positions 3’ and 4’ resulted in increased cytotoxicity [47]. Thus, some authors have reported that the toxic effects of piceatannol are even more potent than those exhibited by trans-resveratrol, pterostilbene, or trans-stilbene-oxide [23,47,48,49,50,51].

In contrast, very few authors reported tan absence of cytotoxic effects after exposure to piceatannol in different leukemic cell lines at concentrations up to 50 µM after 24 h and 48 h, and up to 100 µM after 48 h of exposure [37,38,48]. Moreover, high concentrations of piceatannol (400 µM) showed a non-cytotoxic effect in murine melanoma cell lines [42].

In order to complete these results, morphological assays were performed by these authors (Table 2). The results showed that this compound induced apoptosis in a dose-dependent manner causing cell shrinkage, chromatin and nuclear condensation, and apoptotic bodies. Low concentrations (1 µM) of the compound can induce spherical apoptotic beads after 48 h of exposure in SK-Mel-28 cancer cells [40]. In contrast, it is interesting to point out that no study has been performed to evaluate the effects of piceatannol in the morphology of non-cancerous cells. 

The results of the in vitro cytotoxicity studies carried out with pterostilbene are shown in (Table 3). A comparison between all cytotoxic studies is difficult since the exposure conditions, cell lines, and endpoints differed. In general, most of the authors indicated that this stilbene shows cytotoxic effects in several cell models at different conditions in a range of 25–100 µM. The lowest IC_50_ value reported was 1.81 µM in SOSP-9607 cells after 24 h of exposure measured by the MTT assay [49]. 

On the other hand, although the IC_50_ values for non-cancerous cell lines could not always been calculated, a reduction in cell viability was observed after exposure to pterostilbene. The percentage of cell viability of Chang human liver cells was reduced to 75% after exposure to 100 µM of this stilbene after 24 h [50]. Moreover, a very important decrease in cell proliferation was observed in CRL-158 human placenta cells exposed to pterostilbene at concentrations of 40 and 80 µM resulting in reductions of 61.8% and 72.2% as compared to the control [29]. 

Pterostilbene is expected to be a potent cytotoxic agent since the introduction of one or more methoxy groups into the stilbene structure was previously observed to increase the cytotoxicity of stilbene derivatives [43]. This agrees with the results obtained by several authors comparing the effect of this stilbene with other structurally modified stilbenes, observing that pterostilbene exhibits more potent effects than resveratrol, piceatannol, trans-3,5,4’-trimethoxystilbene, and 3,5,4’-triacetylstilbene [45,64,68,84,87]. 

Moreover, the cytotoxicity study of pterostilbene has been completed with several morphological assays (Table 4). The methods used for this purpose were fluorescence microscopy using acridine orange (AO) and ethidium bromide (EB), staining with 4, 6-diamidino-2-phenylindole (DAPI) or Hoechst 33342, and electron microscopy. Low concentrations of pterostilbene caused morphological changes indicating the induction of apoptosis in different cells. The SOSP-9607 cell line treated for 24 h with 1 µM of pterostilbene showed loss of confluence [49]. Moreover, MCF-7 cells exposed to 5 µM for 24 h suffered shrinkage, membrane and cytoplasmic blebbings and chromatin condensation [72]. Moreover, in the case of pterostilbene, no morphological assays were performed on non-cancer cell lines. 

The cytotoxic studies performed with ε-viniferin are reported in Table 5. In general, concentrations ranging from 10–200 µM of ε-viniferin caused a significant decrease in the cell viability of cancer cells in a time- and concentration-dependent manner. Low IC_50_ values for trans-ε-viniferin were found in HL-60, HepG2, and AGS carcinoma cell lines with values of 5.6 µM ± 1.4, 7.7 µM ± 0.2, and 9.3 µM ± 0.3, respectively [93]. Moreover, ε-viniferin cytotoxicity in non-cancerous cells has also been demonstrated [30,32,46]. Chowdhury et al. (2005) [32] stated that the 50% cytotoxic concentrations of (-)-ε-viniferin in human oral cell lines HGF, HPC, and HPLF were 111 µM, 146 µM, and 94 µM, respectively, which is of interest since ε-viniferin concentrations of 100–200 µM were used in most of the studies performed. Moreover, only 49.9 µM of this compound was required to inhibit the growth by half in MRC-5 normal human lung cells [46]. Higher concentrations were needed to induce toxicity in the non-transformed human hepatocyte cell line HH4, and the IC_50_ values obtained after 24 and 48 h of exposure were 192.7 µM and 177.9 µM, respectively.

This compound’s lack of cytotoxicity has also been demonstrated in various cancer and non-cancer cell lines (SW480, L1210, K562, HCT116, PC12, HepG2, and Chang cells) [37,99,100,101]. It is interesting to indicate that, although different exposure times have been evaluated (24–96 h), the absence of toxic effects in some cases may be due the low concentrations studied (10, 30, and 50 µM) [37,99,100].

Furthermore, since ε-viniferin is a resveratrol dimer, it can possess a trans or cis configuration. Moreover, this stilbene is a chiral molecule that can cause dextrorotation (−) and levorotation (+). Most of the authors did not specify which ε-viniferin configuration was evaluated. Among those who reported the configuration, trans-ε-viniferin was the most studied was because it is more stable than the cis configuration. The effects of both isomers have been evaluated by Kim et al., (2002) [94]. Concentrations up to 100 µM of both cis and trans isomers induced similar cytotoxic effects in C6, HepG2, HeLa, MCF-7, and HT-29 cancer cell lines after 70 h of exposure. Moreover, the IC50 values obtained in all cell lines were comparable for both configurations [94]. Furthermore, (−)-ε-viniferin was also selected by several authors, but only Chang et al. (2017) [102] evaluated the cytotoxic effects of (+)-ε-viniferin, hindering the comparison between both configurations.

The morphological changes produced by ε-viniferin have been reported by four authors, as far as we know. The main results of these studies are described in Table 6. After exposure to 100 µM for 24 h, and 95 µM and 130 µM for 48 h different cancer cells (HL-60 and C6) suffered chromatin condensation, nuclear fragmentation and contraction [96,104]. Thus, it seems that a prolonged exposure to this compound does not result in more damage. Moreover, low concentrations of ε-viniferin (30 µM and 60 µM) for 48 and 72 h did not produce apoptotic changes in SW480 and HT144 cancer cell lines [99,103], evidencing that concentrations higher than 60 µM are needed to induce ultrastructural damage. Finally, it should be emphasized that nuclear staining with Hoechst was the only technique performed in these assays and there were no studies evaluating the effect of ε-viniferin in non-cancerous cells.

## 3. Genotoxicity in In Vitro Studies Performed with Stilbenes

In general, very few in vitro studies have been performed to investigate the potential genotoxic effects and the DNA damage produced by piceatannol, pterostilbene, or ɛ-viniferin. In fact, there is no research whose main objective has focused on this aspect. Specifically, only 11, 10, and 3 studies of piceatannol, pterostilbene and ε-viniferin, respectively, are related to this topic (Table 7).

The Guidance for submission for food additive evaluations of the EFSA Panel on Food Additives and Nutrient Sources added to Food [15,116] reported that the mutagenic and genotoxic potential of new additives must be assessed in view of the adverse consequences of genetic damage to human health. To address genotoxicity studies, EFSA guidelines indicate two mandatory tests for all food additives, the Ames test and the in vitro mammalian cell micronucleus test. These tests meet the basic requirements to cover the three genetic endpoints with the minimum number of tests.

Among all the studies conducted with piceatannol, only Makena and Chung (2007) [110] performed one of the two tests required by the EFSA for the evaluation of its genotoxic potential. These authors carried out the Ames test using only one *Salmonella typhimurium* strain (TA102), out of the 5 strains recommended by the EFSA. They showed a non-mutagenic effect at 50 µg/plate of piceatannol in the presence and absence of metabolic activation (rat liver S9 mix). However, the main objective of their work was not to evaluate the potential genotoxicity of piceatannol, but to demonstrate the antimutagenic effect of this compound against the mutations induced by benzidine at 50, 100, and 200 µg/plate in the TA102 strain. In addition to this work, there are also two reports that use the comet assay to evaluate the DNA damage produced by piceatannol in different cell lines. The comet assay is an efficient tool to measure single and double-strand DNA breaks at the cellular level [85]. Thus, Azmi et al. (2005) [109] stated that piceatannol produced more damage than resveratrol in the DNA of human peripheral lymphocytes at 10, 20, and 50 µM of piceatannol in the presence of Cu (II); however, no data for piceatannol without Cu (II) was reported. On the other hand, the other study only focused on demonstrating the protective effect of this compound. Ovesná et al. (2006) [35] showed a decrease in the DNA damage produced by H_2_O_2_ in L1210, K562, and HL-60 cell lines at 1, 2.5, and 5 µmol/L. Moreover, other techniques such as flow cytometry, western blot analysis and electrophoresis have indicated that piceatannol produces DNA damage, electrophoresis being the most widely used assay [32,34,53,60,112,113]. In general, different studies have demonstrated that piceatannol produces fragmentation in a dose-dependent manner in some cell lines such as HL-60, HSC-2 [32], U937 [53], A549, and HepG2 [112] by electrophoresis. To date, no in vitro micronucleus assays have been performed with piceatannol as required by the EFSA to ensure its safety as far as we know. 

In relation to genotoxicity and DNA damage studies performed with pterostilbene, different techniques such as the micronucleus test, comet assay, electrophoresis, western blot analysis, and the TUNEL assay have been performed. Rossi et al. (2013) [114] stated that pterostilbene does not produce micronuclei at concentrations of 20, 40, and 80 µM in CHO-K1 cells after 3 h of exposure. Furthermore, they confirmed that this stilbene reduced basal DNA damage present in untreated cells under these same conditions by the comet assay. Moreover, the latter authors observed that 80 µM of pterostilbene can reduce the oxidative damage produced by H_2_O_2_ as measured by the comet assay but it did not show a protective effect against the induction of micronuclei produced by H_2_O_2_. Furthermore, antimutagenic effects of pterostilbene against 4-nitroquinoline-N-oxide have been detected by the comet assay at 50 µM [67]. Similar to piceatannol reports, most of the studies performed with pterostilbene used electrophoresis. Different authors have evidenced that pterostilbene can produce DNA fragmentation in different cell lines such as HeLa [74,82], MCF-7 [62,72], PC3 [62], and MOLT4 [69] at different concentrations (from 10 to 200 µM) and exposure periods (from 12 to 48 h). Despite being required by the EFSA, no Ames test studies have been performed with this substance thus far.

Among the three stilbenes studied in this review, ɛ-viniferin has been the least studied in regard to its genotoxic and DNA-damaging potential. Kim et al. (2002) [94] performed the Ames test in order to evaluate the antimutagenic potential of ɛ-viniferin. They used the TA100 strain of *Salmonella typhimurium*, exhibiting its antimutagenic potential at a concentration of 35.2 g/plate. However, no information about the mutagenic potential of the substance was reported for this assay. In addition, more recent studies have demonstrated that ε-viniferin produces DNA damage in the A431 cell line by the comet assay [115] and the C6 cell line by the TUNEL assay [104].

As the results showed, none of the three reviewed substances have been assessed by both in vitro tests (Ames test and micronucleus assay) required by the EFSA as the first step in genotoxicity testing. Moreover, most of these studies have been carried out on cancer cell lines and their main objective was not to study the genotoxic potential of these stilbenes as required by the EFSA for all food additives to ensure consumer safety. In this sense, the DNA damage has been investigated as a possible mechanism of cytotoxicity against cancer cells.

Taking into account these results, we consider it necessary and scientifically relevant to evaluate the performance of the in vitro genotoxicity assays and the DNA damage caused by these stilbenes prior to their use in the food industry. 

## 4. Toxicological In Vivo Studies Performed with Stilbenes

Studies focused on assessing the toxicity of substances using in vivo models are necessary to guarantee the safety of their use. In this sense, in vivo toxicity studies of piceatannol, pterostilbene, and ε-viniferin in rodents with potential application in the food industry (novel foods, food additives, etc.) are very scarce, and none have fulfilled the assessment required by the EFSA [15,116]. These studies compromise genotoxicity and other toxicity studies such as subchronic and carcinogenicity studies, etc. [103]. It has only been in recent years that studies have been performed to assess the protective effect of these substances against stress and disease in rodents [117,118,119,120]. 

With respect to piceatannol, as far as we know, only two authors have evaluated its potential toxic effect. Kiliç (2019) [118] showed that albino mice administered a dose of 4 mg/kg/day IP for 7 days did not show significant differences in biochemical parameters such as superoxide dismutase, catalase, and malonyldialdehyde as compared to the control group. There was no observable nuclear signal of rabbit monoclonal antibody against proliferating cell nuclear antigen or hepatic DNA damage in the treated group. With respect to the results of the histological analysis, apoptotic hepatocytes were rarely observed in animals exposed to piceatannol. Moreover, Shi and Fu (2019) [120] showed that 10 mg/kg/day of piceatannol administered orally via gastric gavage did not induce testicular toxicity. Additionally, beneficial effects such as a marked improvement in mRNA- and protein-expression levels of Nrf2 and its regulated genes and proteins were observed in rats.

The first study that investigated the safety profile of pterostilbene was conducted by Ruiz et al. (2009) [121]. They demonstrated that mice exposed to pterostilbene during 28 days at a dose up to 3000 mg/kg/day caused no mortality during the experimental period. Histopathologic examination and evaluation of biochemical parameters also revealed no alterations regarding organ weight or clinical signs. However, the red blood cell number and hematocrit increased after polyphenol administration as compared to the control group (Ruiz et al., 2009). Later, Riche et al. (2013) [122] assessed the toxicity of pterostilbene in mice after IV administration of 30 mg/kg/day for 23 days. Even at this high dose, pterostilbene was found to be pharmacologically safe as its administration was accompanied by no systemic or organ related toxicity. Moreover, these authors evaluated the long-term safety of pterostilbene administration in a randomized double-blind placebo-controlled trial in humans [122]. They reported that daily doses from 100 mg to 250 mg in adults with hyperlipidemia did not produce a significant adverse drug reaction on hepatic, renal, or glucose markers, with pterostilbene being well-tolerated twice daily. The data available in animal and human models suggests that this compound does not have significant toxic effects. However, the existing information is not adequate to justify the positive effects of this compound in humans after prolonged administration beyond the recommended dietary dose [119]. To our knowledge, no in vivo studies about the safety profile of ε-viniferin were described in the scientific literature. In this sense, it is imperative to perform clinical animal research and human trials to address the safety of ε-viniferin after acute and chronic administration prior to its industrial use. 

Taking into account all these facts, further research should include study designs aimed to investigate the safety of these stilbenes in in vivo models. More studies are needed which focus on genotoxicity, subchronic, and chronic toxic effects, etc. to portray the comprehensive safety aspects and to reinforce its human relevancy and market prospects.

## 5. Conclusions

Considering the increasing interest in stilbenes as additives in the food industry, toxicological assays are needed to assure their safety. The present review describes the available data on the cytotoxic, mutagenic, and genotoxic aspects of piceatannol, pterostilbene, and ε-viniferin. Their cytotoxic effects depend on the cell lines used, assays performed, and exposure conditions. In general, most of the authors stated that these compounds exhibit toxic effects not only in cancer cells but in non-cancer cell lines. Moreover, the DNA damage induced by these compounds has been demonstrated by several methods as a possible mechanism of cytotoxicity. However, the in vitro genotoxic potential of piceatannol, pterostilbene, and ε-viniferin has been poorly studied and no studies following EFSA guidelines were performed. The largest gap in the toxicity assessment of these compounds is the lack of in vivo studies, since most of the authors have evaluated their beneficial properties but have not evaluated their in vivo toxicity. Thus, in order to guarantee the safe use of piceatannol, pterostilbene, and ε-viniferin, more studies are needed such as toxicokinetic, genotoxicity, subchronic, chronic, and carcinogenicity assays, etc. to fulfill the EFSA’s recommendations. 

## Figures and Tables

**Table 1:** In vitro cytotoxicity studies performed with piceatannol.

Pure Stilbene	Experimental Model	Assays Performed	Exposure Conditions	Main Results	Reference
Piceatannol	BJAB cells	LDH activity	25, 50, 75, and 100 µM for 4 h	The stilbene at concentrations ≤ 100 µM did not reduce cell viability, indicating that the membrane disrupting effect does not play a role in their death-inducing potency.	[51]
Piceatannol	Caco-2 and HCT-116 cells	Crystal violet	12.5, 25, 50, 100, and 200 µM for 24, 48, and 72 h	A steady decrease in cell number was observed in a dose- and time-dependent manner. After the exposure of 200 µM for 72 h, the growth rate of cells decreased 60% ± 3.2% in Caco-2 cells and 58.3 ± 3.1% in HCT-116 cells.	[44]
Piceatannol	U266 and 2F7 cells	XTT assay	50 µM for 24 h	Piceatannol displayed no cytotoxicity in any of the cell lines.	[48]
Piceatannol	SK-Mel-28 cells	MTT assay	25, 50, and 100 µM for 96 h	The stilbene was rendered unstable only 4 h after its addition without an apparent effect on the cell cycle after 48 h of assay.	[40]
Piceatannol	SK-Mel-28 cells	Cell viability by flow cytometry	1–100 µM for 4–48 h	Cell viability decreased with increasing concentrations and incubation time.	[41]
Piceatannol	HGF, HPC, HPLF, HSC-2, HSC-3, HSG, and HL-60 cells	MTT assay	0–1000 µM for 24 h	The four tumor cell lines (HSC-2, HSC-3, HSG, and HL-60) were more sensitive to the stilbene than the three normal cell lines (HGF, HGC, and HPLF). The CC50 values were 367 µM for HGF, 414 µM for HPC, >1000 µM for HPLF, 63 µM for HSC-2, 232 µM for HSC-3, 373 µM for HSG, and 11 µM for HL-60 cells.	[32]
Piceatannol	HL-60 cells	CC-108 microcellcounter	0–100 µM for 72 h	Significant decrease of cell viability at 10 µM. Piceatannol was more cytotoxic than resveratrol. The IC_50_ was set at 9.1 µM ± 0.28	[47]
Piceatannol	L1210, K562, andHL-60 cells	TBET	0–500 µM for 24 h	<20 µM and <10 µM concentrations caused cytotoxicity in L1210 and HL60 cells. The cytotoxic effect was lower in K562 cells.	[35]
Piceatannol (purity > 99%)	RAW264.7 cells	MTT assay	0–50 µM for 48 h, with and without stimulation of zymosan	Cytotoxic effect was significant starting at 30 µM. This effect was attenuated to a significant extent by a cotreatment with zymosan.	[52]
Piceatannol	B16 cells	MTT assay	5, 100, 200, and 400 µM for 24 h	No cytotoxicity. Cell viability was 99.8, 98.7, 95.3, and 90.1% at 5, 100, 200, and 400 µM, respectively.	[42]
Piceatannol (purity > 99%)	RAW 264.7, A–431, 10ScNCr/23, and CCR-CEM cells	TBET Cell proliferation studies using a hemocytometer	0–50 µM for 24 h 10, 30, and 50 µM for 200 h	RAW 264.7 cells were more sensitive to piceatannol than other stilbenes (trans-resveratrol, trans-pterostilbene, and trans-stilbene-oxide). The IC_50_ was set at 1.30 µM ± 0.12. In the cell proliferation studies, there was no affection of 10ScNCr/23 cells at ≤50 μM, whereas an inhibitory effect was observed in RAW 264.7 and A431 cells at 50 μM, and in CCR-CEM cells at ≥10 μM.	[23]
Piceatannol	HL-60 cells	CC-108 microcellcounter	3.125, 6.25, 12.5, and 25 µM for 72 h	The IC_50_ value was 14 µM after 3 days of incubation.	[33]
Piceatannol	U937 cells	TBET	0–80 µM for 48 h	The 48 h treatment reduced cell viability in a concentration-dependent manner.	[53]
Piceatannol	T24 and HT1376 cells	XTT assay	0.5, 2.5, 5, and 10 µM for 48 h	Dose-dependent effect. At 48 h, the maximum effect on proliferation inhibition was observed at 10 µM in both cells.The IC50 values were 3.9 µM in T24 cells and 4.6 µM in HT1376 cells.	[54]
Piceatannol (from Vitis amurensis)	L1210, K362, and HCT116 cells	MTT assay	0–50 µM for 48 h	No cytotoxic effect was observed. The IC_50_ was not found and set at >50 µM.	[37]
Piceatannol	C6 cells (proliferating and growth arrested)	Protein content (Lorry method)	1–100 µM for 72 h in proliferating cells and 24 h in growth-arrested cells	A cytotoxic effect at low micromolar concentrations was recorded in growth-arrested cells. The IC_50_ value in proliferating cells was 28 µM ±4 and in growth-arrested cells was 20 µM ± 2.	[55]
Piceatannol	U937 cells	MTT assay	20, 40, 60, and 100 µM for 24 h	The cells showed a concentration-dependent and time-dependent decrease in cell viability. A reduction in cell viability of approximately 48% was observed after treatment with 5 µM for 24 h.	[56]
Piceatannol	LoVo and LoVo/doxorubicincells	SRB assay	20, 40, 60, 80, and 100 µM for 72 h	In sensitive cells (LoVo), the effect of piceatannol and its derivative (trans-3, 5, 3’, 4’-tetracetoxystilbene) was more toxic than in resistant cells. After exposure to 100 µM for 72 h, the reduction of viability was approximately 50% in LoVo cells and 15% in Lovo/doxorubicin-resistant colon cells.	[43]
Piceatannol	THP-1, HL-60, and U937 cells	MTT assay	10, 20, 30, 40, and 50 µM for 24 h	Treatment with piceatannol resulted in a dose-dependent inhibition of cell viability.	[34]
Piceatannol	HL-60 and HepG2 cells	MTT assay	10–200 µM for 24, 48, and 72 h	A high inhibition was found after treatment with 100–200 µM for 24, 48, and 72 h in HL-60 cells. No significant effect on HepG2 cell growth at the doses and times used.	[24]
Piceatannol	K562 cells	MTT assay	0–100 µM for 48 h	No cytotoxicity was recorded for the concentrations assayed.	[38]
Piceatannol (purity > 99%)	LNCaP, Du145, andPC3M cells	MTT assay	1–100 µM for 6 days	The growth inhibitory effects found were cell specific. The IC_50_ was 31.7 µM in LNCaP cells, 23.2 µM in Du145 cells, and 34.6 µM in PC3M cells.	[45]
Piceatannol	OV2008, C13, A2780s, A2780cp, OVCAR-432, and SkOV-3 cells	MTS assay	10 µM alone and in combination with 10 µM cis-diamminedichloroplatinum for 24 h in all cell lines 10 µM alone and in combination with 10 µM cis-diamminedichloroplatinum for 48 h in OV2008	Piceatannol reduced cell viability in all tested cell lines and enhanced the cytotoxic effects of diamminedichloroplatinum in OV2008, A2780s, and OVCAR-432, concluding that p53 status is a determinant of piceatannol action. A time dependent decrease was observed in OV2008 viability after 48 h of exposure. Moreover, an additive effect with cis-diamminedichloroplatinum was determined. The IC_50_ value of piceatannol for 48 h in OV2008 cells was 29.1 µM.	[25]
Piceatannol	Undifferentiated neural stem cells	MTT assay	1–20 µM for 72 h	No effect was observed at 2.5 µM or less. The IC50 was 13.5 µM.	[57]
Piceatannol (purity > 99%)	WM266-4 and A2058 cells	MTT assay	0–200 µM for 36 h	The growth of both cells was inhibited in a dose-dependent manner. The IC_50_ was 29.4 µM in WM266–4 and 15.6 µM in A2058 cells.	[39]
Piceatannol	NCIH-522 cells	WST-8 assay	10, 30, 50, 80, and 100 µM for 24, 48, and 72 h	The stilbene suppressed proliferation in a dose- and time- dependent manner. The IC_50_ was set at 53, 23, and 17 µM for 24, 48, and 72 h, respectively.	[58]
Piceatannol (purity > 99%)	SW1990 and PANC-1 cells	CCK-8 assay	1, 10, 20, 40, 100, and 200 µM for 72 h	Cell proliferation was inhibited in a dose-dependent manner.The IC_50_ values were 30.69 µM and 21.82 µM for SW1990 and PANC-1 cells, respectively.	[59]
Piceatannol (purity ≥ 98%)	MRC-5, AGS, SK-MES-1, and J82 cells	MTT assay	0–100 µg/mL for 72 h	No cytotoxic effect was observed in non-cancerous cells. The IC_50_ was set at >100 µM in MRC-5 cells, 44.4 µM ± 3.2 in AGS, 31.3 µM ± 2.1 in SK-MES-1 cells, and 27.7 µM ± 1.4 in J82 cells.	[46]
Piceatannol	MOLT-4 cells	NRU assay	0.05, 15, 25, 50, and 100 µM for 48 h	A reduction in cell viability was observed in a concentration-dependent manner. Incubation with piceatannol for 6–8 h led to a significant increase in the number of cells in the sub-G1 fraction, indicating apoptotic DNA degradation. The IC_50_ value was 24.8 µM.	[60]
Piceatannol	HUVEC cells	MTT assay and LDH activity	3–100 µM for 48 h	No effect on cell viability was found up to 30 µM.	[28]
Piceatannol (purity ≥ 98%)	HeLa cells	MTT assay	0–250 µM for 48 h	The stilbene inhibited cell proliferation in a dose- dependent manner. The IC_50_ was 375.20 µM.	[61]

Abbreviations used: 10ScNCr/23 (mouse macrophages); 2F7 (human lymphoma cell line); A2058 (human melanoma cell line); A2780cp (human ovarian cancer cell line); A2780s (human ovarian cancer cell line); A431 (human epidermoid carcinoma cell line); AGS (human gastric adenocarcinoma cell line); B16 (murine melanoma cell line); BJAB (human lymphoma cells); C13 (human ovarian endometrioid adenocarcinoma cell line); C6 (rat glioma cell line); Caco-2 (human colon adenocarcinoma cells); CC_50_ (50% cytotoxic concentration); CCK (cell counter kit); CCR-CEM (human tumor-derived human T cell line); Du145 (human prostate carcinoma cell line); HCT116 (human colon carcinoma cell line); HeLa (human cervix epithelioid carcinoma cell line); HepG2 (human liver adenocarcinoma cell line); HGF (human gingival cell line); HL-60 (human promyelocytic leukemia cell line); HL-60R (human promyelocytic leukemia cell lines); HPC (human pulp cell line); HPLF (human periodontal cell line); HSC-2 (human squamous cell carcinoma cell line); HSC-3 (human tongue squamous carcinoma cell line); HSG (human submandibular gland carcinoma cell line); HT1376 (human bladder cancer cell line); HUT78 (human lymphoma cell line); HUVEC (human umbilical vein endothelial cell line); inhibitory mean concentration (IC_50_); J82 (human bladder cancer cell line); K362 (human cancer cell line); K562 (human erythroleukemia cell line); K562-ADR (human leukemia cell line); L12110 (mouse lymphoma cell line); LDH (lactate dehydrogenase); LNCaP (human prostate adenocarcinoma cell line); LoVo (human colon adenocarcinoma cell lines); MOLT-4 (human lymphoma cell line); MRC-5 (human lung fibroblasts cell line); MTS ((3-(4,5-dimethylthiazol-2-yl)-5 (3-carboxymethoxyphenyl)- 2-(4-sulfophenyl)-2H- tetrazolium salt); MTT (3-(4,5-dimethylthiazol-2-yl)-2,5-diphenyltetrazolium bromide); NCIH-522 (human lung cancer cell line); NRU (neutral red uptake); OV2008 (human ovary endometrioid adenocarcinoma cell line); OVCAR-432 (human ovarian cancer cell line); PANC-1 (human pancreas adenocarcinoma cell line); PC3M (human prostate cancer cell line); RAW 264.7 (mouse macrophages ); SK-MEL-28 (human melanoma cell line); SK-MES-1 (human lung cancer cell line); SkOV-3 (human ovarian cancer cell line); SRB (sulforhodamine B); SW1990 (human pancreas adenocarcinoma cell line); T24 (human bladder epithelial cancer cell lines); TBET (trypan blue dye exclusion test); THP-1(human leukemia cell line); U266 (human myeloma cell line); U937 (human lymphoma cell line); WM266-4 (human melanoma cell line); WST-8 (2-(2-methoxy-4-nitrophenyl)-3-(4-nitrophenyl)-5-(2,4-disulfophenyl)-2H-tetrazolium, monosodium salt); XTT (2,3-bis-(2-methoxy-4-nitro-5-sulfophenyl)-2H-tetrazolium-5-carboxanilide.

**Table 2:** In vitro morphological studies performed with piceatannol.

Pure Stilbene	Experimental Model	Assays Performed	Exposure Conditions	Main Results	Reference
Piceatannol	SK-Mel-28 cells	Hoechst 33258 staining	1 µM for 8–48 h	Bright spherical beads could be seen in apoptotic cells. Apoptotic cells increased about 6-fold with respect to the control after 48 h.	[41]
Piceatannol	HL-60 cells	Hoechst 33258 staining and PI double staining.	5, 10, 20, and 40 µM for 24 h	A dose-dependent increase of apoptotic cells was observed. After incubation with 40 µM, 96% showed early signs of apoptosis.	[33]
Piceatannol	U937 cells	DAPI staining	10, 20, 40, and 60 µM for 48 h	Nuclei with chromatin condensation and the formation of apoptotic bodies were observed in the cells treated with piceatannol in a concentration-dependent manner.	[53]
Piceatannol	THP-1 cells	Light microscopy	10, 20, 30, 40, and 50 for 24 h	Cell shrinkage occurred at concentrations higher than 30 µM.	[34]
Piceatannol	HL-60 and HepG2 cells	Hoechst 33258 staining and PI double staining	50–200 µM for 24–72 h	A time-dependent increase of apoptotic cells was observed, the HL-60 being cells more sensitive.	[24]
Piceatannol	OV2008 cells	Hoechst 33258 staining	10 µM for 24 h	Induction of apoptosis causing nuclear condensation and fragmentation was found.	[25]

Abbreviations used: DAPI (4, 6-diamidino-2-phenylindole); HepG2 (human liver adenocarcinoma cell line); HL-60 (human leukemia cell line); OV2008 (human ovary endometrioid adenocarcinoma cell line); PI (propidium iodide); SK-MEL-28 (human melanoma cell line); THP-1 (human leukemia cell line); U937 (human lymphoma cell line).

**Table 3:** In vitro cytotoxicity studies performed with pterostilbene.

Pure Stilbene	Experimental Model	Assays Performed	Exposure Conditions	Main Results	Reference
Pterostilbene	HL-60, HL-60R, K562, K562-ADR, and HUT78 cells	TBET	1–100 µM for 48 h	Pterostilbene exhibited a similar inhibiting effect and dose response curve in all cell lines. The IC_50_ values obtained were 35 µM ± 7 in HL-60 cells, 24 µM ± 3 in HUT78, 10 µM ± 3 in K562 cells, 40 µM ± 3 in HL60-R cells, and 12 µM ± 2 in K562-ADR cells.	[36]
Pterostilbene (purity > 97%)	RAW 264.7 cells	MTT assay, TBET, and cell proliferation studies with hemocytometer	0–50 µM for 24 h in MTT and trypan blue assays 10, 20 and 30 µM for 200 h in cell proliferation studies	Maximum inhibition was found from 20 to 30 µM. Cells cultured from > 72 h with < 10 µM were significantly different from the controls. The IC_50_ for MTT was 8.33 µM ± 0.88 and for TBET was 4.03 µM ± 0.12.	[23]
Pterostilbene isolated from Pterocarpus marsupuim	MCF-7 and PC3 cells	MTT assay	0–100 µM for 24 h	Inhibition of cell growth was clearly observed from 40–80 µM. The IC_50_ values were 65.6 µM in MCF-7 and 74.3 µM in PC3 cells.	[62]
Pterostilbene (purity > 96%)	T24 and T24R cells	MTT assay	50, 75 and 100 µM for 72 h	Growth decreased in both cell lines in a concentration- and time-dependent manner. The IC_50_ values for 48 h were 66.58 µM ± 1.84 in T24 cells and 77.95 ± 0.44 µM in T24R cells.	[63]
Pterostilbene (purity > 98%)	HepG2 and Chang cells	MTT assay	3.125, 6.25, 12.5, 25, 50, and 100 µM for 24 h	A concentration-dependent decrease of cell viability in both cell lines was observed. However, no IC_50_ could be obtained.	[50]
Pterostilbene	HCT116, HT-29 and Caco-2 cells	MTT assay	0–100 µM for 48 h	Cancer cells were more sensitive to pterostilbene than resveratrol, Caco-2 being the least. The IC50 values were 12 µM in HCT116 cells, 15 µM in HT-29 cells, and 75 µM in Caco-2 cells.	[64]
Pterostilbene	SK-MEL, KB, BT-549, SK-OV-3, Vero, and LLC-PK11 cells	NRU assay for solid tumor cells and TBET for non-cancerous cells	0–25 µg/mL for 48 h	Moderate cytotoxicity was observed. No IC_50_ value was obtained.	[65]
Pterostilbene	HT-29 cells	MTT assay	0–100 µM for 24 h	No cytotoxic effect was observed. The highest concentration tested only reduced cell viability by 20.17% ± 0.82.	[66]
Pterostilbene (purity ≥ 90%)	CHO-K1 cells	MTT assay	20, 40, 60, 80, and 100 µM for 24 h	No cytotoxicity was recorded at lower concentrations; however, the growth inhibitory effect on cells was significant at 100 µM.	[67]
Pterostilbene	A375, A549, HT-29, and MCF7 cells	Countess Automated Cell Counter	0–100 µM for 24, 48, and 72 h	Exposure to pterostilbene reduced tumor cell number in a concentration-, time-, and in a cell line-dependent way. Pterostilbene was more cytotoxic than resveratrol. The IC_50_ was set at 60.3 µM in HT-29 cells, 44 µM in MCF7 cells, 14.7 µM in A375, and 28.6 µM in A549 cells.	[68]
Pterostilbene	K562 cells	MTT assay	0–100 µM for 48 h	Pterostilbene exhibited significant cytotoxicity while other stilbenes had slight cytotoxic effects. The IC_50_ value was 67 µM.	[38]
Pterostilbene	MOLT4 cells	NRU assay	0–100 µM for 48 h	A dramatic decrease in cell viability was shown. The estimated concentration required to inhibit cell growth by 90% was 44 µM.	[69]
Pterostilbene	MCF-7 andBcap-37 cells	MTT assay	0–150 µM for 24 h, 48 h, and 72 h	Inhibition of cell proliferation was recorded in a time- and dose-dependent manner. The IC_50_ values ranged from 50–100 µM for both cell lines and exposure times (24, 48, and 72 h).	[70]
Pterostilbene (purity ≥ 99%)	LNCaP, Du145, and PC3M cells	MTT assay	1–100 µM for 6 days	Growth inhibition was reported for all tested cells. Pterostilbene displayed the highest cytotoxicity among piceatannol, resveratrol and two of its derivatives in PC3M cells. The IC_50_ values were 22.8 µM in LNCaP cells, 20.8 µM in Du145, and 17 µM in PC3M cells.	[45]
Pterostilbene	SOSP-9607 cells	MTT assay	1, 2 and 4 µM for 12 h, 24 h, and 36 h	Cell growth was inhibited in a dose- and time-dependent manner. The IC_50_ value at 24 h was 1.81 µM.	[49]
Pterostilbene	A431 cells	MTT assay	15, 30, and 60 µM for 24 h	No cytotoxic effect was recorded.	[71]
Pterostilbene (purity ≥ 98%)	A549 and A549 docetaxel resistant cells	MTT assay	50, 75, and 100 µM for 24 h, 48 h, and 72 h	A significant decrease in the growth of both cell lines in a concentration- and time-dependent manner was reported.	[26]
Pterostilbene	MCF-7, T47D, PC-3, NCIH-522, HepG2, PA-1, and LNCaP cells	MTT assay	0–100 µM for 24 h	Dose-dependent inhibition was found. MCF7, T47D, and HepG2 were more sensitive to pterostilbene. The IC_50_ was 65 µM ± 0.42 in MCF-7, 69 µM ± 1.58 in T47D, 75 µM ±3.55 in PC-3, 85 µM ± 2.64 in NCIH-522, 73 µM ± 1.81 in HepG2, 120 µM ± 2 in PA-1, and 70.4 µM ± 4.39 in LNCaP cells.	[72]
Pterostilbene	Caco-2 cells	SRB assay and LDH activity	5, 10, 25, 40, 50, 60, 75, and 100 µM for 48 h in both assays and 72 h in SRB assay	Cells exposed to concentrations from 40–100 µM for 48 h exhibited significantly decreased cellular density and an increase in LDH release. At 72 h, all concentrations tested showed significant inhibition of cell proliferation.	[73]
Pterostilbene	HeLa cells	MTT assay	5–160 µM after 24 h, 48 h, and 72 h	Rapid increase in the inhibition rate showing an “S” shape curve. At 80 µM, inhibition was 53.1% after 48 h.	[74]
Pterostilbene (purity ≥ 98%)	SAS and OECM-1 cells	MTT assay	0–40 µM for 24 h and 48 h	Cell viability substantially decreased in a time-dependent manner in both cell lines.	[75]
Pterostilbene	Caco-2, HCT116, and CRL-158 cells	SRB assay	0–1000 µM for 72 h in cancer cells and 40 and 80 µM in CRL-158 cells	Both cells suffered significant inhibition of viability, the non-cancerous cells being the most sensitive. The IC_50_ values were 31.2 µM ± 0.42 in Caco-2 and 84.4 µM ± 1.14 in HCT116 cells.	[29]
Pterostilbene	MCF-7 and MCF-7 CD44+/CD24- cells	TBET	0–75 µM for 72 h	The effect of pterostilbene was more potent in MCF-7 CD44+/CD24-. The IC_50_ recorded was 25 µM in MCF-7 CD44+/CD24-.	[76]
Pterostilbene	NU-DUL-1, OCI-LY8, U2932, SUDHL-4, DB, and TMD8 cells	CCK-8 assay	12.5, 25, 50, 75, and 100 µM for 48 h	Cell proliferation was significantly inhibited in a dose-dependent manner but not in a time-dependent way in SUDHL-4, DB, and NU-DUL-1 cells from 12.5 to 100 µM for 24 h, 48 h, and 72 h.	[77]
Pterostilbene	H929, ARP-1, OCI-MY5, and RPMI-8226 cells	CCK-8 assay	10, 20, 30, 40, and 50 µM for 24 h, 48 h, and 72 h	Decrease of cell viability in a dose- and time-dependent manner. The IC_50_ values obtained for 72 h were 15.37 µM ± 0.98 in H929 cells, 26.15 µM ± 3.6 in ARP-1 cells, 43.36 µM ± 4.46 in OCI-MY5, and 23.58 µM ± 0.41 in RPMI-8226.	[78]
Pterostilbene (purity > 97%)	RAW 264.7 cells	MTT assay and TBET	3, 10, 20, and 30 µM for 24 h and 48 h	Concentration-dependent toxicity was observed. Among all the stilbenes studied, pterostilbene was the most cytotoxic followed by piceatannol and resveratrol. The IC_50_ values for the MTT assay were 20.7 µM for 24 h and 19 µM for 48 h. Moreover, the IC_50_ values for TBET were 4 µM for 24 h and 3.6 µM for 48 h.	[79]
Pterostilbene	BT-20 and MDA-MB-468 cells	MTT assay	10, 20, 40, and 80 µM for 48 h	A dose-dependent inhibition of cell proliferation was consistently observed.	[80]
Pterostilbene	PC9 and A549 cells	CCK-8 assay	20, 40, and 60 µM for 24 h and 48 h	Inhibition of cell viability in a dose- and time-dependent manner. The IC_50_ values were 50.9 µM for 24 h and 27.35 µM for 48 h in PC9 cells, and 52.01 µM for 24 h and 24.12 µM for 48 h in A549 cells.	[81]
Pterostilbene	HeLa cells	MTT assay	5, 25, 50, 100, 200, and 400 µM for 24 h and 48 h	Dose- and time-dependent cytotoxic effects were recorded. The IC_50_ values were 101.2 µM for 24 h and 65.9 µM for 48 h.	[82]
Pterostilbene	Cisplatin-resistant CAL 27 cells	MTT assay	5, 10, 25, 50, 75, and 100 µM for 24, 48, and 72 h	A time- and concentration-dependent decrease in cell number was reported. The IC_50_ values were 78.26 µM ± 4.33 for 24 h, 48.04 µM ± 3.68 for 48 h, and 20.65 µM ± 4.88 for 72 h.	[83]
Pterostilbene	HeLa cells	WST-1 assay	10–20 µM for 24 h	A dose-dependent effect was shown. Pterostilbene exhibited higher cytotoxicity than resveratrol at the same concentrations. The IC_50_ value was 42.3 µM.	[84]
Pterostilbene	BV-2 cells	MTT assay	1, 5, 10, and 20 µM for 24 h	Pterostilbene did not affect the viability of BV-2 cells.	[85]
Pterostilbene	Daudi and K562 cells	CCK-8 assay	0–100 µg/mL for 24 h, 48 h, and 72 h	A time- and dose-dependent decrease in cell viability was observed. The IC_50_ was 6.87 µM ±1.02 in Daudi cells and 7.05 µM ±1.14 in K562 cells.	[86]
Pterostilbene	TC1 cells	WST-1 assay	5–100 µM for 72 h	Concentration-dependent cytotoxicity was observed. Pterostilbene was more cytotoxic than resveratrol. The IC_50_ was 15.61 µM	[87]
Pterostilbene	NCIH-520 and NCIH-226 cells	MTT assay	1.56, 3.13, 6.25, 12.5, 25, and 50 µM for 24 h and 48 h	Cytotoxicity was observed for all the cells in a dose-dependent manner. H520 cells were more sensitive than the H226 cells. The IC_50_ was 47.7 µM ± 5.3 for 24 h and 31.4 µM ± 4.6 for 48 h in H520 cells and >50 µM for 24 h and 44.3 µM ± 3.7 for 48 h in H226 cells.	[88]
Pterostilbene	CCD-18-Co, HCT116, SW480, and HT-29 cells	MTT assay	10, 20, 40, 60, 80, and 100 µM for 24 h	Pterostilbene did not affect the viability of normal colon CCD-18-Co cells, but it reduced the viability of HT-29, SW480, and HCT116 cells.	[89]
Pterostilbene (purity > 98%)	HaCat and JB6 cells	MTT assay	3.75, 7.5, 15, 30 µM and 60 µM for 24 h	Only an exposure of 60 µM decreased cell viability in a significant manner in both cell lines.	[90]
Pterostilbene	MIA PaCa-2 and gemcitabine-resistant MIA PaCa-2 cells	MTT assay	5, 10, 25, 50, and 75 µM for 48 and 72 h	Suppression of cell proliferation in a time- and dose-response manner. Similar EC_50_ values were obtained after 72 h of exposure for both cell lines (41.8 µM in MIA PaCa-2 cells and 42 µM in gemcitabine-resistant MIA PaCa-2 cells).	[91]
Pterostilbene (purity ≥ 98%)	RAW 264.7 and HCEC cells	MTT assay	2.5, 5, 7.5, 10, 12.5, 15, 17.5, and 20 µM for 24 h	Dose-dependent cytotoxic effects toward both cell lines were shown. No IC_50_ was reported.	[92]
Pterostilbene	A498, ACHN, and HK-2 cells	MTT assay and LDH assay	5, 10, 20, 50, and 100 µM for 24 h, 48 h, and 72 h in the MTT assay10, 20, and 50 µM for 24 h for LDH	Potent cytotoxic effects were recorded in renal cancer cells but not in non-cancerous renal cells. The cytotoxicity followed a dose- and time-dependent pattern.	[93]

Abbreviations used: A431 (human epidermoid carcinoma cell lines); A498 (human renal carcinoma cell lines); A549 (human alveolar adenocarcinoma cells); ACHN (human renal adenocarcinoma cell line); ARP-1 (human myeloma cell line); Bcap-37 (human breast adenocarcinoma cell line); BT-20 (human breast cancer cell lines); BT-549 (human breast carcinoma cell line); BV-2 (murine microglial cell line); Caco-2 (human colon adenocarcinoma cells); CAL 27 (human tongue carcinoma cell line); CCD-18-Co (human colon cell lines); CCK (cell counter kit); Chang (non-malignant Chang’s liver cells); CHO-K1 (hamster ovary cell lines); CRL-158 (human placental cell line); Daudi (human lymphoma cell lines); DB (human lymphoma cell line); Du145 (human prostate carcinoma cell line); H929 (human myeloma cell line); HaCat (human epithelial cell line); HCEC (human corneal epithelial cell lines); HCT116 (human colon carcinoma cell line); HeLa (human cervix epithelioid carcinoma cell line); HepG2 (human liver adenocarcinoma cell line); HK-2 (human kidney cell line); HL-60 (human promyelocytic leukemia cell line); HL-60R (human promyelocytic leukemia cell lines); HT-29 (human colon adenocarcinoma cell line); HUT78 (human lymphoma cell line); inhibitory mean concentration (IC50); JB6 (mouse epithelial cell line); K562 (human leukemia cell line); K562 (human lymphoma cell lines); K562-ADR (human leukemia cell line); KB (human oral epidermoid carcinoma cell line); LDH (Lactate dehydrogenase); LLC-PK11(pig kidney epithelial cells); LNCaP (human prostate adenocarcinoma cell line); MCF-7 (human breast adenocarcinoma cell line); MDA-MB-468 (human breast adenocarcinoma cells); MIA PaCa-2 (pancreas carcinoma cell line); MOLT4 (human leukemia cells); MTT (3-(4,5-dimethylthiazol-2-yl)-2,5-diphenyltetrazolium bromide); NCIH-226 (human lung cancer cell lines); NCIH-520 (human lung cancer cell line); NRU (neutral red uptake); NU-DUL-1 (human lymphoma cells); OCI-LY8 (human lymphoma cells); OCI-MY5 (human myeloma cell line); OECM-1 (human oral cancer cells); PA-1 (human ovarian teratocarcinoma cell line); PC3 (human prostate adenocarcinoma cell line); PC3M (human prostate cancer cell line); PC9 (human lung adenocarcinoma cell line);RAW 264.7 (mouse macrophages); RPMI-8226 (human myeloma cell line); SAS (human oral cancer cells); SK-MEL (human melanoma cell line); SK-OV-3 (human ovary adenocarcinoma cell line); SOSP-9607 (human osteosarcoma cell line); SRB (sulforhodamine B); SUDHL-4 (human lymphoma cells); SW480 (human colon adenocarcinoma cell line); T24 (human bladder epithelial cancer cell lines); T24R (chemoresistant human bladder epithelial cancer cell line after long-term nicotine exposure); T47D (human breast cancer cell line); TBET (trypan blue exclusion test); TC-1 (mouse lung epithelial cell line); TMD8 (human lymphoma cells); U2932 (human lymphoma cells); Vero (monkey kidney fibroblast cell line); WST-1 (Water Soluble Tetrazolium salt-1).

**Table 4:** In vitro morphological studies performed with pterostilbene.

Pure Stilbene	Experimental Model	Assays Performed	Exposure Conditions	Main Results	Reference
Pterostilbene isolated from Pterocarpus marsupuim	MCF-7 cells and PC3 cells	AO and EB by fluorescence microscopy and scanning electron microscopy	MCF-7 cells were exposed to 65.6 µM for 24 h and PC3 cells to 74.3 µM for 24 h	Cells exposed to the stilbene showed chromatin condensation, distorted surface morphology, loss of intercellular attachments and membrane blebbing.	[62]
Pterostilbene (purity > 96%)	T24 and T24R cells	DAPI staining, AO, electron microscopy, and phase contrast microscopy	100 µM for 24 h, 48 h, and 72 h	Cytoplasm and cell surface had granular appearances in exposed cells. Autophagic vacuoles and autolysosomes were also found. Induction of acidic vesicular organelles was also reported.	[63]
Pterostilbene	MCF-7 and Bcap-37 cells	DAPI staining and electron microscopy	50 µM for 24 h	Treated cells showed a foamy appearance and nuclear condensation. Floating cells were also observed.	[70]
Pterostilbene	SOSP-9607 cells	Phase contrast microscopy	1, 2, and 4 µM for 24 h	A decrease in cellular attachment was observed.	[49]
Pterostilbene (purity ≥ 98%)	A549 and A549 docetaxel resistant cells	DAPI staining and microscopic observation	100 µM for 48 h using microscopic observation and 50, 75, and 100 µM for 48 h using DAPI staining	Formation of vacuoles, condensed and fragmented nuclei were found in both cell types after 48 h.	[26]
Pterostilbene	MCF-7 cells	Phase contrast microscopy and AO and EB by fluorescence microscopy	5, 15, 30, and 50 µM for 24 h	Apoptosis induction was evidenced by cell shrinkage and loss of confluence in a dose- dependent manner. Chromatic condensation and loss of cytoplasmic membrane integrity were also found.	[72]
Pterostilbene	HeLa cells	Hoechst 33342 staining, AO, and EB by fluorescence microscopy	80 µM for 48 h	Granular nuclei and nuclear fragmentation were also observed.	[74]
Pterostilbene (purity ≥ 98%)	SAS and OECM-1 cells	DAPI staining, AO, and microscopic observation	0–40 µM for 24 h	Both cell lines treated with the stilbene induced the formation of vacuoles in the cytoplasm, condensed nuclei, and acidic vesicular organelles in a concentration- and time-dependent manner.	[75]
Pterostilbene	MCF-7 and MCF-7 CD44 +/CD24- cells	Microscopy observation (400x)	100 µM for 6 h	Cell necrosis, such as membrane injury and bleb formation, were found.	[76]
Pterostilbene	PC9 and A549 cells	Phase contrast microscopy	20, 40, and 60 µM for 24 h	Significant cell shrinkage and a decreased cellular attachment rate were reported.	[81]
Pterostilbene	HeLa cells	AO and EB by fluorescence microscopy	25, 100, and 200 µM for 48 h	Pterostilbene-treated cells showed cellular shrinkage, detached from one another and from the substratum, membrane blebbing, nuclear fragmentation, and chromatin condensation.	[82]
Pterostilbene	Cisplatin-resistant CAL 27 cells	AO, Monodans yleadaverine LysoTracker Red, Cathepsin B, Hoechst 33342, and phase contrast microscopy	5, 10, 25, 50, 75, and 100 µM for 24 h, 48 h, and 72 h for phase contrast microscopy 25, 50, and 75 µM for 24 h for the other tests	An increased number of acidic vesicular organelles, accumulation of the autophagic vacuole marker and suppressed lysosome activity were observed. Moreover, DNA condensation was shown to occur at 25, 50, and 75 µM.	[83]
Pterostilbene	HeLa cells	Brightfield analysis	40 µM for 24 h	Apoptotic blebbing was observed. Pterostilbene reduced cell numbers more markedly than resveratrol when cells were exposed to the same concentration.	[84]
Pterostilbene	TC1 cells	Brightfield analysis	5–100 µM for 72 h	Cytoplasmic blebbing was reported after 48 h at <10 µM.	[87]
Pterostilbene	HCT116, SW480, and HT29 cells	DAPI staining and phase contrast microscopy	40 µM for 24 h	Apoptotic bodies, DNA fragmentation and cell shrinkage	[89]
Pterostilbene	NCIH-520 and NCIH-226 cells	Brightfield analysis	1.56, 3.13, 6.25, 12.5, 25 and 50 µM for 24 h and 48 h	Apoptotic morphological changes, cell shrinkage and cytoplasmic blebbing. H520 cells were more sensitive than NCIH-226 cells.	[88]
Pterostilbene	A498 and ACHN cells	Phase contrast microscopy	10, 20 and 50 µM for 24 h and 48 h	A decreased number of cells and a reduction in cell-cell contact were observed in cancer cells in a dose-dependent manner.	[92]

Abbreviations used: A549 (human alveolar adenocarcinoma cells); ACHN (human renal adenocarcinoma cell line); AO (Acridine orange); Bcap-37 (human breast adenocarcinoma cell line); DAPI (4,6-diamidino-2-phenylindole); A498 (human kidney carcinoma cell line); CAL 27 (human tongue carcinoma cell line); EB (ethidium bromide); HeLa (human cervix epithelioid carcinoma cell line); HCT116 (human colorectal carcinoma cell line); HT29 (human colon adenocarcinoma cell line); NCIH-226 (human lung cancer cell lines); NCIH-520 (human lung cancer cell line); MCF-7 (human breast adenocarcinoma cell line); OECM-1 (human oral cancer cells); PC3 (human prostate adenocarcinoma cell line); PC9 (human lung adenocarcinoma cell line); SAS (human oral cancer cells); SOSP-9607 (human osteosarcoma cell line); SW480 (human colorectal carcinoma cell lines); TC-1 (mouse lung epithelial cell line); T24 (human bladder epithelial cancer cell lines); T24R (chemo resistant human bladder epithelial cancer cell line after long-term nicotine exposure).

**Table 5:** In vitro cytotoxicity studies performed with *trans*-ε- viniferin.

Pure Stilbene	Experimental Model	Assays Performed	Exposure Conditions	Main Results	Reference
*Trans*-ε-viniferin and *cis*-ε-viniferin (from *Paeonia lactiflora*)	C6, HepG2, HeLa, MCF-7, and HT-29 cells	MTT assay	0–100 µM for 70 h	Cytotoxicity was reported in all cell lines tested, although *trans*- and *cis*- ε-viniferin were markedly cytotoxic in C6 and HeLa cells. The IC_50_ values for *trans*-ε-viniferin were: 18.4 µM in C6 cells, 74.3 µM in HepG2 cells, 20.4 µM in HeLa cells, 44.8 µM in MCF-7 cells, and 88.4 µM in HT-29 cells. The IC_50_ values for *cis*-ε-viniferin were: 20.1 µM in C6 cells, 76.2 µM in HepG2 cells, 21.5 µM in HeLa cells, and 47.2µM in MCF-7 cells, and 90.2 µM in HT-29 cells.	[94]
ε-viniferin (from vine-shoots)	WSU-CLL cells	TBET	0–100 µM for 24, 48, and 72 h	A concentration- and time-dependent decrease in cell viability was observed, ε-viniferin˂ resveratrol. The inhibition of cell multiplication was paralleled by a decrease in DNA synthesis. The IC50 at 72 h was 60 µM.	[95]
ε-viniferin	HL-60 cells	MTT assay	10–200 µM for 24 h	Cell viability decreased in a dose-dependent manner. The IC50 was 33 µM.	[96]
(-)-ε-viniferin	HCF, HPC, HPLF, HSC-2, HSC-3, HSG, and HL-60 cells	MTT assay in adherent cells and TBET in non-adherent cells	0–1000 µM for 24 h	The four tumor cell lines (HSC-2, HSC-3, HSG, and HL-60) were more sensitive to the stilbene than the three normal cells (HCF, HPC, and HPLF). The CC_50_ values were 111 µM for HCF cells, 146 µM for HPC cells, 94 µM for HPLF cells, 42µM for HSC-2 cells, 84 µM for HSC-3 cells, 110 µM for HSG cells, and 31 µM for HL-60 cells.	[32]
(-)-ε-viniferin	P-388 cells	MTT assay	0–100 µM for 48 h	ε-viniferin moderately inhibited the cells in comparison to hopeaphenol which exhibited a greater effect. The IC_50_ found was 18.1 µM ± 0.7.	[97]
ε-viniferin	HepG2 cells	TBET	30 µM for 24, 48 and 72 h. 1, 5, 10, 30, 60, and 100 µM for 48 h	At 60 µM, ε-viniferin completely inhibits cell proliferation. After 48 h, the toxicity potential of ε-viniferin was lower than resveratrol. The IC_50_ for 48 h was 58.4 µM.	[98]
ε-viniferin	SW480 cells	TBET (Coulter Counter) and MTT assay	30 µM for 24, 48, 72, and 96 h in trypan blue assay. 3, 30, 60, and 100 µM for 48 h in coulter counter	Cells exposed to the stilbene grew similarly to the control. Reduced growth rate and percentage of cell inhibition. In the MTT assay, no inhibition of cell proliferation was recorded.	[99]
*Trans*-ε-viniferin (from Vitis amurensis)	L1210, K562, and HCT116 cells	MTT assay	0–50 µM for 48 h	No cytotoxic activity was recorded. Therefore, the IC_50_ was assumed to be above 50 µM.	[37]
ε-viniferin glucoside	PC12 cells	MTT assay	0–10 µM for 24 h	Cell viability was not significantly affected in any exposure to the stilbene.	[100]
(-)-ε-viniferin	HepG2 and Chang cells	MTT assay	1.56–200 µg/mL for 72 h	No cytotoxic effect was found in either cell.	[101]
ε-viniferin	VSMCs	MTS assay	10, 20, and 30 µM for 48 h	The antiproliferative rate of ε-viniferin at 20 µM was significantly higher than that of resveratrol at both 20 and 30 µM.	[30]
(+)-ε-viniferin (from Ampelopsis brevipedunculata)	RAW264.7 cells	MTT assay	1, 5, and 10 µM for 12 h	(+)-ε-viniferin dramatically reduced cell viability to 60% after the exposition of 10 µM. No IC_50_ was obtained.	[102]
ε-viniferin	HT-144 and SK-MEL-25 cells	MTT assay and TBET	25–200 µM for 24, 48, and 72 h	A decrease in cell survival in a time- and dose-dependent manner was observed in both cell lines. The IC_50_ for 48 h was 60 µM.	[103]
ε-viniferin	C6 cells	WST-1 assay	95 and 130 µM for 12, 24, and 48 h	A decrease in cell proliferation was reported. This reduction was significant at all concentrations and times tested.	[104]
*Trans*-ε-viniferin	MRC-5, AGS, SK-MES-1, and J82 cells	MTT assay	0–100 µg/mL for 72 h	Cytotoxic effects were observed in all tested cell lines. The IC_50_ values were 49.9 µM ± 3 in MRC-5 cells, 42.6 µM ± 1.7 in AGS cells, 78.8 µM ± 3.3 in SK-MES-1 cells, and 56.7 µM ± 1.2 in J82 cells.	[46]
*Trans*-ε-viniferin	Mouse primary co-culture of astrocytes and neurons	CellTiter 96 ^®^ Aqueous	1, 5, 10, 20, 50, and 100 µM for 72 h	A significant decrease in cell viability was observed at exposures of 50 and 100 µM.	[105]
ε-viniferin	Caco-2 cells	MTT and NRU assays	1.56, 3.12, 6.25, 12.5, 25, 50, and 100 µM for 24 h	A dose-dependent decrease in cell viability was observed at 25 µM. ε-viniferin was slightly more toxic than resveratrol in Caco-2 cells.	[106]
*Trans*-ε-viniferin	COLO 205, HT-29, HepG2, AGS, and HL-60 cells	MTT assay	0–100 µg/mL for 48 h	Dose-dependent cytotoxicity was reported, with a potent effect observed in HL-60 cells. The IC_50_ values were: 85.5 µM ± 8.1 in COLO205 cells, 13.9 µM ± 0.1 in HT-29 cells, 7.7 µM ± 0.2 in HepG2 cells, 9.3 µM ± 0.3 in AGS cells, and 5.6 µM ± 1.4 in HL-60 cells.	[93]
*Trans*-ε-viniferin (from Vitis vinifera)	HepG2, Hep3B, and HH4 cells	Crystal violet assay	0–200 µM for 24, 48, and 72 h	Cell number decreased in a dose- and time-dependent manner, being more cytotoxic in Hep3B cells. In HH4 cells, higher concentrations were needed to induce toxicity. The IC_50_ values obtained were the following: - HepG2 cells: 140 µM ± 39.7 (24 h), 103.8 µM ± 19.2 (48 h), 94.8 µM ± 28.3 (72 h) - Hep3B cells: 108.1 µM ± 31.8 (24 h), 73.9 µM ± 17.3 (48 h), 63.1 µM ± 10.8 (72 h) - HH4 cells: >200 µM (24 h), 192.7 µM ± 21.1 (48 h), 177.9 µM ± 20.5 (72 h).	[107]
*Trans*-ε-viniferin	HepG2 and Caco-2 cells	MTS assay, NRU, and protein content	0–100 μg/mL for 24 and 48 h	Both cell lines exposed to ε-viniferin exhibited a time-dependent decrease for all the endpoints studied The EC_50_ values were: HepG2: 28.28 ± 2.15 24 h and 17.85 ± 3.03 for 48 h. Caco-2 cells: 36.72 ± 3.01 for 24 h and 20.63 ± 1.25 48 h.	[108]

Abbreviations used: AGS (human gastric adenocarcinoma cell line); C6 (rat glioma cancer cell line); Caco-2 (human colorectal adenocarcinoma cell line); CC_50_ (50% cytotoxic concentration); Chang (non-malignant Chang’s liver cell line); COLO 205 (human colon adenocarcinoma cell line); effective mean concentration (EC50); HCF (human gingival cell line); HCT116 (human colon adenocarcinoma cell line); HeLa (human cervix epithelioid carcinoma cell line); Hep3B (human hepatic cancer cell line); HepG2 (human liver adenocarcinoma cell line); HH4 (non-transformed human hepatocyte cell line); HL-60 (human promyelocytic leukemia cell line); HPC (human pulp cell line); HPLF (human periodontal cell line); HSC-2 (human squamous carcinoma cell line); HSC-3 (human tongue squamous carcinoma cell line); HSG (human submandibular gland carcinoma cell line); HT-144 (human melanoma cell line); HT-29 (human colon adenocarcinoma cell line); inhibitory mean concentration (IC_50_); J82 (human bladder cancer cell line); K562 (human erythroleukemia cell line); L1210 (mouse lymphoma cell line); MCF-7 (human breast adenocarcinoma cell line); MRC-5 (human lung fibroblasts cell line); MTS (3-(4,5-dimethylthiazol-2-yl)-5-(3-carboxymethoxyphenyl)-2-(4-sulfophenyl)-2H-tetrazolium); MTT ((3-(4,5-dimethylthiazol-2-yl)-5 (3-carboxymethoxyphenyl)- 2-(4-sulfophenyl)-2H- tetrazolium salt); NRU (neutral red uptake); P-388 (murine leukemia cell line); PC-12 (rat adrenal gland cancer cell line); RAW 264.7 (mouse macrophages); SK-MEL-25 (human melanoma cell line); SK-MES-1 (human lung cancer cell line); SW480 (human colon adenocarcinoma cell line); TBET (trypan blue dye exclusion test); VSMCs (human vascular smooth muscle cell line); WST-1 (Water Soluble Tetrazolium salt-1); WSU-CLL (human lymphoblastic leukemia cell line).

**Table 6:** In vitro morphological studies performed with ε-viniferin.

Pure Stilbene	Experimental Model	Assays Performed	Exposure Conditions	Main Results	Reference
ε-viniferin	HL-60 cells	Hoechst 33342 staining	100 µM for 24 h	The cell line suffered chromatin condensation, nuclear fragmentation and cell collapse into apoptotic bodies.	[96]
ε-viniferin	SW480 cells	Hoechst 33342 staining	30 µM for 48 h	No apoptotic changes were observed.	[99]
ε-viniferin	HT-144 and SK-MEL-25 cells	Hoechst 33342 staining	60 µM for 48 and 72 h	Increase in nucleus size, condensation, and fragmentation of nuclear chromatin in SKMel-28 cells, but not in HT144 cells.	[103]
ε-viniferin	C6 cells	Hoechst 33342 staining	95 and 130 µM for 48 h	Condensation of chromatin structure and slight contraction in the nuclear membrane.	[104]

Abbreviations used: C6 (rat glioma cancer cell line); HL-60 (human promyelocytic leukemia cell line); HT-144 (human melanoma cell line); SK-MEL-25 (human melanoma cell line); SW480 (human colon adenocarcinoma cell line).

**Table 7:** In vitro genotoxicity and DNA damage studies performed with piceatannol, pterostilbene and ε-viniferin.

Pure Stilbene	Experimental Model	Assays Performed	Exposure Conditions	Main Results	Reference
Piceatannol	BJAB cells	Flow cytometric determination of hypodiploid DNA	0, 25, 50, 75, 100, and 125 µM for 4 h	Dose-dependent DNA fragmentation was observed.	[51]
Piceatannol	Human peripheral lymphocytes	Comet assay	0, 10, 20, and 50 µM for 30 min in the presence of Cu (II)	DNA breakage was detected. The effect exerted by piceatannol was more potent than that of resveratrol and *trans*-stilbene.	[109]
Piceatannol	HL-60 and HSC-2 cell lines	Electrophoresis	10, 20, and 40 µM to HL-60 or 320 µM to HSC-2 for 6 or 4 h, respectively	Piceatannol induced DNA fragmentation in a dose-dependent manner.	[32]
Piceatannol	L1210, K562, and HL-60 cell lines	Comet assay	Pretreatment of 0.625, 1, 2.5, or 5 µM for 24 h	A decrease in DNA damage was produced by H_2_O_2_ after piceatannol exposure.	[35]
Piceatannol	Salmonella typhimurium TA102 strain	Ames test	50 µg/plate 200, 100, and 50 µg/plate	No mutagenicity was reported in the presence and absence of rat liver S9 mix. The stilbene exhibited antimutagenic activity in the presence of rat liver S9 mix. Moreover, it moderately inhibited the effect of the mutations produced by benzidine.	[110]
Piceatannol	U937 cells	Electrophoresis	0, 10, 20, 40, or 60 µM for 48 h	Dose-dependent DNA fragmentation was detected after piceatannol exposure.	[53]
Piceatannol	THP-1 cells	Electrophoresis	10 µM for 24 h	Tumor necrosis factor-related apoptosis-inducing ligand (TRAIL). DNA breakage was reported only in the presence of 20 ng/mL.	[34]
Piceatannol	Du145 cells	Western blot	0, 10, and 25 µM for 72 h	Piceatannol caused DNA damage supported by increased phosphorylated histone H2AX.	[111]
Piceatannol	A549, HepG2, and MCF7 cells lines	Electrophoresis	20, 40, or 60 µg/mL of piceatannol encapsulated with nanoparticles (chitosan and poly lactic acid) for 24 h	DNA fragmentation was observed in all cell lines. In A549 and HepG2 cell lines, the effect was dose-dependent.	[112]
Piceatannol	THP-1, HL-60, U937 and K562 cell lines	Electrophoresis	25 or 50 µM for 24 h	After the piceatannol exposure, DNA breaks were observed in all cell lines tested.	[113]
Piceatannol	MOLT-4 cells	Electrophoresis	45.5 µM for 12, 24, and 48 h	DNA fragmentation was detected, with the most potent effect observed after 12 h of exposure.	[60]
Pterostilbene	MCF-7 and PC3 cell lines	Electrophoresis Comet assay	65.6 µM in MCF-7 and 74.3 µM in PC3 for 24 h	DNA damage was observed with both techniques. The comet assay indicated that MCF-7 cells were more sensitive than the PC3 cell line.	[62]
Pterostilbene	MOLT4 cells	Electrophoresis	44 µM for 12, 24, and 48 h	Pterostilbene induced DNA breakage at all exposure times.	[69]
Pterostilbene	CHO-K1 cells	Standard and modified alkaline comet assay. Cytokinesis block micronucleus assay	40 and 80 µM for 3 h 40 and 80 µM for 3 h plus 20 min with H_2_O_2_ 20, 40, and 80 µM for 3 h 20, 40, and 80 µM for 3 h plus 20 min with H_2_O_2_	In non-treated cells, basal damage decreased in a dose-dependent manner. At 80 µM, pterostilbene exhibited a protective effect, reducing the DNA oxidative damage more significantly than trimethoxystilbene and resveratrol. No increase in micronuclei was observed. The stilbene did not protect against H_2_O_2_ oxidative damage.	[114]
Pterostilbene Pterostilbene isothiocyanate conjugate	MCF-7 cells	Electrophoresis	20 µM for 24 h 60 µM for 24 h	DNA breakage was reported. The conjugate produced DNA fragmentation.	[72]
Pterostilbene	HeLa cells	Electrophoresis	80 and 120 µM for 24 h	Pterostilbene induced DNA damage.	[74]
Pterostilbene	HepG2 cells	Comet assay	50 µM plus 1 µM of 4NQO for 4 h in co-exposure Pre-exposure at 50 µM for 4 h, and plus 1 µM of 4NQO for 4 h	In both assays performed, pterostilbene exhibited antimutagenic effects.	[67]
Pterostilbene	H929 cells	Western blot	10, 20, and 40 µM for 24 h	DNA breakage was indicated by the increase in the marker ƴ-H2AX.	[69]
Pterostilbene	HeLa cells	Electrophoresis	25, 10, or 200 µM for 48 h	After pterostilbene exposure, DNA damage was detected in a dose-dependent manner.	[82]
Pterostilbene	CAR cells	TUNEL assay	50, 75, and 100 µM for 48 h	The TUNEL assays indicated that DNA fragmentation was induced by pterostilbene.	[83]
Pterostilbene	ACHN and A498 cell lines	Immunofluorescence analysis Western blot	10, 20, and 50 µM for 24 h	Pterostilbene increased ƴ-H2AX, indicating DNA damage.	[92]
ε-viniferin	*Salmonella typhimurium* TA100 strain	Ames test	35.2 µg/plate	The stilbene exhibited an antimutagenic effect in a dose-dependent manner against the mutations produced by N-methyl-N-nitro-N-nitrosoguanidine.	[94]
ε-viniferin contained in grapevine-shoot extract	A431 cells	Standard and modified alkaline Comet assay	≥25 µg/mL for 1 h	An increase in DNA strand breaks was reported.	[115]
ε-viniferin	C6 cells	TUNEL assay	95 and 130 µM for 12, 24, and 48 h	ε-viniferin produced DNA damage in a dose-dependent manner.	[104]

Abbreviations used: A-431 (human epidermoid carcinoma cells); A498 (human renal carcinoma cell lines); A549 (human alveolar adenocarcinoma cells); ACHN (human renal adenocarcinoma cell line); BJAB (human lymphoma cells); CAR (Cisplatin-resistant human oral cancer cells); CHO-K1 (hamster ovary cell lines); C6 (rat glioma cell line); Du145 (human prostate carcinoma cell line); HeLa (human cervix epithelioid carcinoma cell line); HepG2 (human liver adenocarcinoma cell line); HL-60 (human promyelocytic leukemia cell line); H929 (human myeloma cell line); HSC-2 (human squamous cell carcinoma cell lin4e); K562 (human leukemia cell line); L12110 (mouse lymphoma cell line); MCF-7 (human breast adenocarcinoma cell line); MOLT4 (human leukemia cells); 4NQO (4 -nitroquinoline-N-oxide); PC3 (human prostate adenocarcinoma cell line); THP-1 (human leukemia cell line); U937 (human lymphoma cell line).

## Data Availability

Not applicable.
